# Self-Healing Systems in Silicon Anodes for Li-Ion Batteries

**DOI:** 10.3390/ma15072392

**Published:** 2022-03-24

**Authors:** Neslihan Yuca, Ilknur Kalafat, Emre Guney, Busra Cetin, Omer S. Taskin

**Affiliations:** 1 Enwair Energy Technologies Corporation, Maslak, Istanbul 34469, Turkey; ikalafat@enwair.com (I.K.); eguney@enwair.com (E.G.); bcetin@enwair.com (B.C.); 2 Department of Electric-Electronic Engineering, Maltepe University, Maltepe, Istanbul 34857, Turkey; 3 Department of Chemical Oceanography, Institute of Marine Science and Management, Istanbul University, Istanbul 34134, Turkey

**Keywords:** self-healing, polymers, silicon anodes, lithium-ion batteries, energy storage

## Abstract

Self-healing is the capability of materials to repair themselves after the damage has occurred, usually through the interaction between molecules or chains. Physical and chemical processes are applied for the preparation of self-healing systems. There are different approaches for these systems, such as heterogeneous systems, shape memory effects, hydrogen bonding or covalent–bond interaction, diffusion, and flow dynamics. Self-healing mechanisms can occur in particular through heat and light exposure or through reconnection without a direct effect. The applications of these systems display an increasing trend in both the R&D and industry sectors. Moreover, self-healing systems and their energy storage applications are currently gaining great importance. This review aims to provide general information on recent developments in self-healing materials and their battery applications given the critical importance of self-healing systems for lithium-ion batteries (LIBs). In the first part of the review, an introduction about self-healing mechanisms and design strategies for self-healing materials is given. Then, selected important healing materials in the literature for the anodes of LIBs are mentioned in the second part. The results and future perspectives are stated in the conclusion section.

## 1. Introduction

The use of composite materials has gradually increased in studies carried out for electronic devices, including sensors, batteries, conductors, solar cells, supercapacitors, electronic skin, and the aviation industry, where technological processes that are important in technology development are rapidly adopted. With the development of technology, the ability of composite materials to adapt to the conditions of their environments and to respond appropriately to these conditions is also important. These materials are called smart materials and develop stimuli in a way that changes their mechanical, electrical, optical, or magnetic properties in response to external stimuli. The production of such smart materials leads to the emergence of research topics such as increasing the durability of use, prolonging their life, and/or reducing the cost of healing, and engineers conduct many studies on these issues. This can be achieved by a perfect mechanism called self-healing in biological systems. In order to apply this mechanism to materials, biological systems are studied and imitated. In this context, studies on new-generation smart materials have created a new research area called self-healing materials, and research in this area continues to progress rapidly. Thanks to this behavior, it is argued that the life and reliability of materials that are defective due to production or damaged as a result of an external effect can be increased, and thus healing costs can be reduced [1,2,3,4,5,6,7].

**Figure 1:**
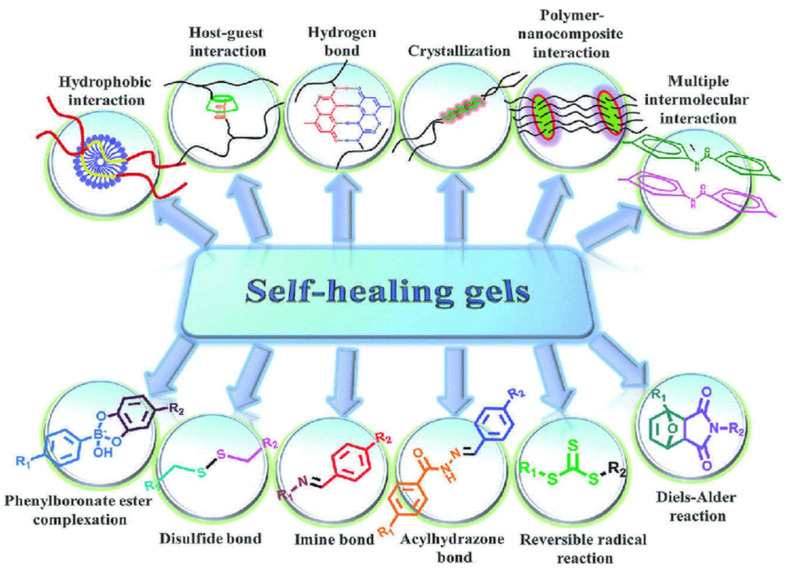
Self-healing systems. Reprinted with permission from ref. [7]. Copyright 2012 Elsevier.

Self-healing can be defined as the ability of a material to heal (recover/repair) damages autonomously without any outside intervention. Many general terms are used to describe such properties in materials, such as self-healing, autonomic healing, and autonomic healing. When self-healing properties are added to man-made materials, the self-healing action often cannot be performed without an external trigger. There are several systems used to impart self-healing ability to materials (Figure 1). These systems can be classified into two main groups: capsule-based healing (bead, fiber, and/or vascular type and mechanochemical), which is autonomous, and healing by the action of nonautonomous external stimuli [1,8,9,10,11,12,13].

**Figure 2:**
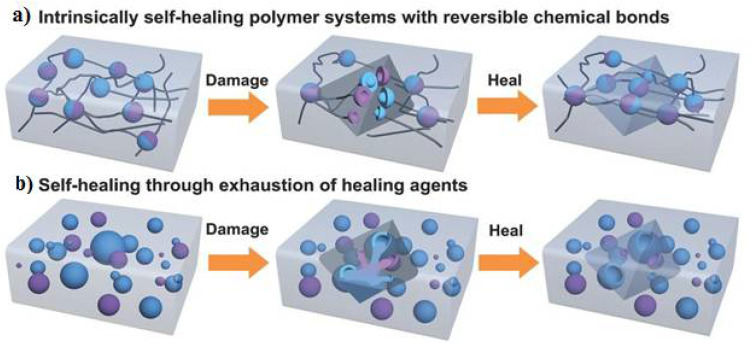
Intrinsically capsulated self-healing systems. Reprinted with permission from ref. [17]. Copyright 2009 Wiley.

Capsule-based healing systems involve microencapsulations and protect micron-sized solid particles, liquid droplets, or gas by isolating them from the external environment with an inert shell. The capsule ensures that the healing agent is retained within the system until a break or crack occurs in the self-healing materials (Figure 2). In capsule-based heal systems, interfacial, in situ, coacidification, and soluble solution encapsulation techniques are among the most basic techniques [1,14,15,16,17].

Other examples of autonomic self-healing systems are fiber and vascular systems. Fiber and/or vascular self-healing systems are designed from fibers (hollow fiber) or hollow reticulated structures. In these systems, the healing agents are stored in the spaces inside the fiber or reticular structures until damage occurs and are released in case of damage. Hollow fibers are used as polymer additives in one, two, and three dimensions. In the region where the crack occurs, the resin and curing agent in the fibers flow into the polymer matrix and polymerize, and as a result, they allow the closure of the crack. Although hollow fibers placed in one dimension seem to be advantageous due to their ease of manufacture, their self-healing capabilities are limited compared to other designs [10,18,19,20,21,22,23].

The other method used to gain self-healing ability is nonautonomous mechanisms and uses latent effects that allow self-healing of damage [24,25,26,27,28]. Supramolecular polymers, which emerged with the combination of polymer science and supramolecular chemistry, have created a novel interdisciplinary research field. These noncovalent interactions in supramolecular systems can be classified as π-π stacking interactions, metal–ligand interactions, ionic interactions, and hydrogen bonds [29,30,31,32].

The large volume changes during the repeated insertion/extraction process of lithium ions can lead to cracking or pulverization of the silicon anodes in the lithium-ion batteries (Li-ion), which reduces the cycle life of the batteries. It has been realized that damages by volume changes in the silicon, abrasion, cutting, breakage, and operational fatigue often produced in practical usage, and degradation over time will result in deterioration of a device’s properties and significant shorting of the device’s life. Fractures and other damages that occur in materials start microscopically, and as the fracture energy cannot be effectively distributed in the structure, the fracture grows and spreads throughout the material. The synthesis of polymers in which damages can be easily controlled or repaired has become very significant [15,33,34,35].

In the last two decades, research interest has been focused on self-healing systems for energy storage devices. Some tremendous reviews have presented the development of self-healing electronic devices, including sensors, supercapacitors, batteries, solar cells, and electronic skin [1,3,17].

In recent years, the “self-healing with smart modifications” approach, which has attracted increasing attention from the scientific community around the world, gives priority to systems that allow the use of new-generation polymeric binders in silicon anodes. Therefore, the self-healing systems in silicon anodes have increased in the past few years [1,7,9]. A comprehensive review of self-healing systems in silicon anodes for LIBs is urgently required. In this regard, we aim to present self-healing materials in silicon anodes and show recent attractive examples of self-healing systems for LIBs. In the first part of the review, an introduction on self-healing systems and design strategies is given. Then, selected important healing materials in the literature for the silicon anodes of LIBs are mentioned in the second part. We hope that the review will provide comprehensive data to attract more attention to self-healing systems in silicon anodes for LIBs.

### 1.1. Various Approaches of Self-Healing Polymers

#### 1.1.1. π-π-Stacking-Interaction-Based Self-Healing Polymers

Although π-π stacking interactions are weaker than hydrogen bonds and ionic interactions, they have an important role in supramolecular systems due to the low probability of their degradation by environmental factors such as humidity. The interactions between aromatic rings of different sizes, shapes, and displacement patterns are called π-π stacking or π-π interactions. Aromatic π interactions first emerged in the early 1980s and have been applied in many fields, especially self-assembly and organic transistors. The interaction usually occurs between the π-deficient electron unit and the π-rich electron unit, and the interaction realizes in mainly two ways: face-to-face stacking and face-to-side stacking. π-π stacking interactions in self-healing supramolecular polymer materials were first obtained by Burattini et al. by combining polyimides containing multiple π-deficient electron acceptor sites and siloxane polymers. In such interactions, the nature of the electron-poor components is critical in terms of interactions, and it also affects the bond strength of the material by playing a role in determining the bond strength of the stack [36,37].

#### 1.1.2. Metal–Ligand-Based Self-Healing Polymers

The compound formed by the coordination of a central atom (M) with different numbers of atoms or groups of atoms called ligands (L) is called a coordination compound or complex. The central atom, ligands, and coordination compound can be neutral or ionic. The central atom is usually a positively charged transition element. Ligands, on the other hand, are anionic or molecular structures and may contain one or more unshared electron pairs. The coordination compound formation reaction can be thought of as a Lewis acid–base reaction, considering that the central atom is an electron pair acceptor and ligands are electron pair donors for joint use [38].

Unlike polymers healed through hydrogen bonds or π-π stacking interactions, the stimulus–response and reversibility of metallopolymers can have better healing performance due to metal–ligand binding resistance.

#### 1.1.3. Ionic-Interaction-Based Self-Healing Polymers

Ionic interactions in polymers are mainly manifested by the formation of ionomers. Ionomers can be defined as polymers in which the volumetric properties are governed by ionic interactions in discrete regions of the material. Since ionomers contain ionic, dipole–dipole, and/or ion–dipole bonds, they also occupy an important place among supramolecular polymer materials. Ionic groups can aggregate together to form a complex. When ionomers crack, self-healing occurs through resilient intermolecular interactions between the ionic groups. In self-healing polymers, the polymer matrix must provide sufficient mobility for the polymer chains so that ionic interactions can take place at the damaged sites, thus allowing the chains to be intertwined and rearranged. In addition, many factors, such as the ionic groups and counter-ions, temperature, degree of neutralization, dielectric constant, and content of ionic groups, also play an important role in the properties of materials that self-heal through ionic interactions [14,39].

#### 1.1.4. Hydrogen-Bond-Based Self-Healing Polymers

Among the various self-healing mechanisms in supramolecular polymers, healing through hydrogen bonding has attracted the attention of many research groups because the hydrogen bonds can be easily separated and reconnected at room temperature, and the recovery properties can be easily adjusted by manipulating the number of hydrogen bonds. Self-healing supramolecular polymers contain both covalent and noncovalent bonds in their structure. The basis of damage to materials is the breaking of chemical bonds. In self-healing materials containing hydrogen bonds, hydrogen bonds are easier to break than covalent bonds. When cracks occur as a result of applying an external force to a supramolecular polymer, multiple free, unbonded hydrogen bonds are formed at the new interfaces. These free hydrogen bonding parts come together and form new hydrogen bonds, allowing the cracks to close and the damaged areas to heal. However, the activity of free hydrogens can continue for a while; the self-healing abilities of the new surfaces will decrease due to the recombination of free hydrogens in the same regions. On the other hand, the reduced self-healing property can be significantly increased by the heat treatment applied to the fracture surfaces [18,40,41].

Most of the work on self-healing constitutes research on understanding and improving mechanisms. In this section, studies on supramolecular healing mechanisms are mentioned, including those of self-healing systems that heal through reversible hydrogen bonding, which is the main subject of this research, are included. Cordier et al. used reversible hydrogen bonds to form supramolecular self-healing rubber. They took advantage of the natural recycling of hydrogen bonds and the bond orientation that allows the chains in the network structure to self-assemble. A mechanical stimulus was needed to initiate the healing of the material obtained by Cordier et al., and the polymer structure was brought together by contacting the damaged surfaces. Thus, hydrogen bonds were allowed to form the reticulated structure. Hydrogen bond formation in this material was provided by aminoethyl imidazolidone and diaminoethyl urea groups, and it was observed that no crystalline region was formed during self-healing. It has been reported that the material produced by this method elongates up to the breaking point with 500% strain. In addition, it has been explained that less than 5% residual stress is seen with the removal of the applied force, and it has the capacity to recover after 300% strain. The visually self-healing test for damage in rubber has been performed by observing the specimens’ healing at room temperature. It has been emphasized that the healed sample can be deformed by up to 200% without breaking with a contact time of 15 min. It was also stated that the ability of the materials to recover decreased as the time elapsed before reassembling the damaged surfaces. With this mechanical intervention, it has been proven by the tests that the healing cycle can be successfully performed many times by contacting the broken or broken parts without using any chemicals [13,41,42,43,44,45].

### 1.2. Effect of Nanoparticle Additive on Self-Healing Properties

Nanoparticle doping has been carried out to increase the healing properties of self-healing systems in the literature. The healing process in nanoparticle-doped polymers does not consist of steps such as breaking or recombining polymer chains. As cracks and defects occur, nanoparticles dispersed in the polymer phase fill the cracked or damaged part. Firstly, Lee et al. combined computer simulation with micromechanics to demonstrate the self-healing effect of nanoparticles in polymers and conducted research on the multilayer composites produced [46]. It has been observed that such polymer–nanoparticle composites actively respond to damage and potentially repeated self-healing of the polymer system as long as the nanoparticles continue to exist in the system. In another publication, they modeled the functionality of applied nanocomposite coatings to heal nanoscale defects on the surface with molecular dynamics and lattice spring simulations. The modeling results show that nanoparticles tend to migrate to the damaged areas with a polymer-induced attraction force, that small particles are more effective at healing the damaged area than large particles, and that those small particles are transported to the damaged area in a shorter time interval. Gupta et al. experimentally proved the transport and aggregation of nanoparticles around cracks in multilayer composite structures in the simulation studies in the literature. In the study, 3.8 nm CdSe/ZnS nanoparticles were embedded in the SiO_2_ layer (50 nm) deposited on the PMMA film (300 nm), and it was observed that the nanoparticles in the fragile SiO_2_ layer were transported to the polymer phase along the crack. It was stated that the transport of nanoparticles depends on the enthalpic and entropic interactions between the PMMA matrix and the nanoparticles. As a result of the TEM analysis applied to the cross-sectional area of the composite material, it was observed that nanoparticles whose surface was modified with fluorescent PEO ligands were deposited on the interface of PMMA and SiO_2_ layers. The role of nanoparticles in the self-healing phenomenon is explained by the stretching and stretching movements of the polymer chains close to the damaged area, and the tendency to decrease the nanoparticle–polymer interaction with the accumulation of nanoparticles in the crack and precrack regions is stated to be the driving force [47]. Bing et al. presented a novel approach providing a composite system to heal the damage of material which was prepared liquid-metal (LM)-mediated spontaneous repairing conductive-additive-free Si anode for a Li-ion battery. The as-prepared nanocomposite of LM/Si showed superior performances as characterized by high capacity utilization (2300 mAh g^−1^, long-term stability (968 mAh g^−1^ after 1500 charge–discharge cycles), and high rate capability (360 mAh g^−1^ at 20 A g^−1^) [48].

## 2. Recently Reported Self-Healing Anode Systems

### 2.1. Physical-Interaction-Based Self-Healing Materials

For self-healing materials that can exhibit reversible properties, there were originally two noncovalent approaches, hydrogen bonding and π-π stacking [2,49]. In addition, Harada et al. applied the host–guest molecular gels for macroscopic self-healing [50]. Nakahata et al. showed supramolecular materials that have self-healing properties and induce a sol–gel phase transition through host–guest interactions provided with poly(acrylic acid) (PAA)-possessing β-CD as a host polymer with PAA-possessing ferrocene as a guest polymer [51]. Kakuta et al. reported supramolecular hydrogels with β-cyclodextrin and adamantane guest molecules mended through a host–guest interaction show self-healing features [52]. Deng et al. synthesized curable crosslinked polymer gels under acidic conditions with reversible covalent acylhydrazone bonds [53]. Matyjaszewski et al. reported trithiocarbonate units activated by external stimuli in their structures for the synthesis of self-healing crosslinked polymers and gels [54,55]. Lehn et al. investigated new Diels–Alder self-healing materials that exhibit structural transformation in the absence of external stimuli [56]. Scaiano et al. showed a DABBF that works as a dynamic covalent bond for autonomous self-healing [57]. Fox et al. reported reinforcement with cellulose nanocrystals to afford a healable nanocomposite material that supramolecular polymer mixture formed through π-π interactions [36]. Ying et al. reported nitrogen-bound urea and its use to make polyureas and poly(urethane-urea) capable of catalyst-free dynamic property change and auto-repair at low temperatures [24]. For the first time, a self-assembled supramolecular gel of metal–ligand and polypyrrole hydrogel with high conductivity and a hybrid gel based on nanostructured polypyrrole was created [38]. Li et al. reported a self-healing network crosslinked by coordination complexes that consisted of ligands via both nitrogen and oxygen atoms of the carboxamide groups under room temperature [28]. Yan et al. demonstrated, for the first time, that a synthetic hydrogel material prepared from polyethylene glycol and polyethyleneimine exhibits self-healing abilities. [58]. Nishimura et al. demonstrated networks of silyl ether linkages incorporated into covalently crosslinked polymer reprocessability [59]. Urban et al. demonstrated that commodity copolymers, such as poly(methyl methacrylate)/n-butyl acrylate (p(MMA/nBA)) and their derivatives, can self-heal upon mechanical damage with key-and-lock commodity self-healing behavior [6]. Zn^2+^ imidazole crosslinks are distributed in a hydrogen-bonded–Diels–Alder dynamic covalent double-crosslinked network [26].

#### 2.1.1. Hydrogen-Bonded Supramolecular Self-Healing

**Figure 3:**
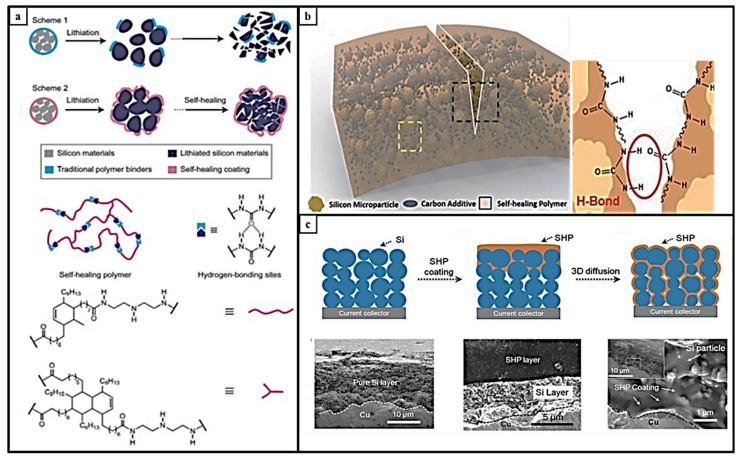
(**a**). Mechanism of the self-healing polymer, reprinted with permission from ref. [62], copyright 2015, *Advanced Energy Materials*. (**b**). Scheme of the self-healing electrode with a homogenous distribution of silicon microparticles and self-healing polymer with hydrogen bond. Reprinted with permission from ref. [63], copyright 2018, Royal Society of Chemistry. (**c**). Scheme of silicon anode with self-healing polymer and SEM images of silicon anode with self-healing polymer coating, reprinted with permission from ref. [62], copyright 2015, *Advanced Energy Materials*.

Phase separation effects at polymeric interfaces are also determinants of self-healing. Kovalenko et al. has used sodium alginate (Algae) as a binder instead of commercial PVdF. In contrast to PVdF, NaAlg contains carboxyl groups that have hydrogen bonds on the oxidized Si surface. This self-healing binder prevents the volume expansion with these bonds during lithiation/delithation. It is the first example of the use of algae as a binder for the Si anode, which exhibited a specific discharge capacity of 1700 mAh g^−1^ after 100 cycles at 4200 mA g^−1^ [60]. The electrode material developed with the self-healing binder proposed by Wang showed a more stable cycling performance with a capacity of up to 2000 mAh g^−1^ 100 cycles at a current density of 0.4 A g^−1^ [61]. Chen et al. provided the 3D spatial distribution of self-healing polymers in silicon nanoparticles with the interaction of hydrogen bonds and healed cracks through the interaction between polymer and silicon, as shown in Figure 3a–c [62]. The fatty acid starting materials were first reacted with diethylene triamine and then subsequently with urea to provide hydrogen bonding end groups at the termination of the fatty acid chains. Kim et al. used a self-healing polymer that serves for minimization of the volume expansion of the silicon. The electrode exhibited a specific discharge capacity of 2100 mAh g^−1^ after 100 cycles at C/10 rate [63].

**Figure 4:**
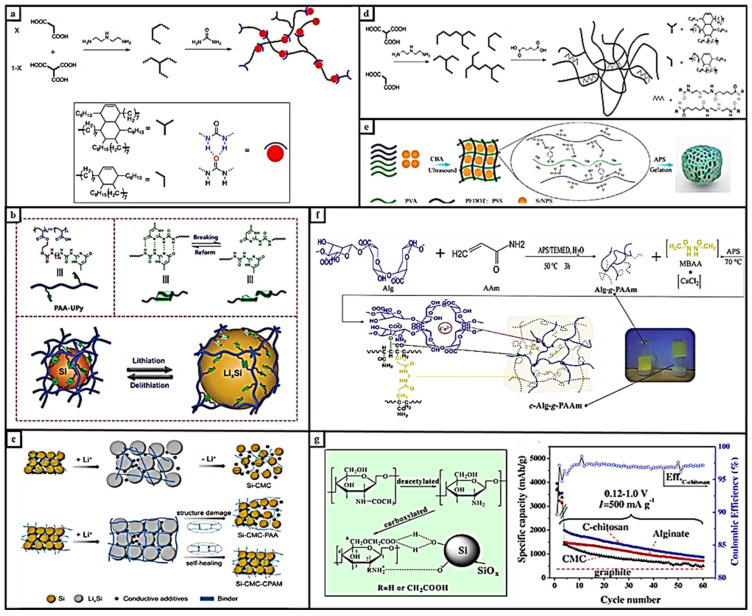
(**a**). The mechanism of the synthesis of self-healing polymers. Reprinted with permission from ref. [29], copyright 2016, *ACS Applied Materials & Interfaces.* (**b**). Structure of PAA–UPy binder and dimers bottomed on quadruple hydrogen bond and the illustration of the large volume expansion of silicon particles, reprinted with permission from ref. [40], copyright 2018, *Advanced Science News*. (**c**). Mechanism with CMC binder crosslinked CMC–PAA binder, and self-healing CMC–CPAM binder, reprinted with permission from ref. [59], copyright 2020, Elsevier. (**d**). Synthesis mechanism of the carboxylic acid functional self-healing polymer, from ref. [65], copyright 2019, *Advanced Materials*. (**e**). Scheme of silicon anode with ultrasonic assisted by PEDOT:PSS binder self-healing conductive hydrogel binder, reprinted with permission from ref. [35], copyright 2020, Elsevier. (**f**). Synthesis of alginic acid and acrylamide-based dual-crosslinking polymers, from ref. [66], copyright 2019, *Journal of Power Sources*. (**g**). C-chitosan/Si nanoparticles surface structure and the capacity–cycle graph of silicon anode with CMC, C-chitosan, and alginate, reprinted with permission from ref. [64], copyright 2014, *Journal of Power Source*.

Yue et al. improved a carboxymethyl chitosan for the Si anode of Li-ion batteries, as shown in Figure 4a. This water-soluble binder-based electrode showed a high specific discharge capacity of 4270 mAh g^−1^ with a first coulombic efficiency of 89% [64]. Self-healing polymers with a high silicon nanoparticle filling cycling stability of trifunctional crosslinked polymer electrodes showed 3–4 mAh cm^−2^ aerial capacity and 140 cycles [29]. Sun et al. reported the flexible carbon/Si foam material that is shown in Figure 4b,c and coated it with a polymer with self-healing functionalization with covalent and hydrogen bonds. The thickness of the self-healing polymer coating on the electrode was affecting the percent of strain and electrochemical capacity of the cell. It was determined that more coating caused rapid capacity fading [65]. The dual-crosslinking polymer shown in Figure 4f heals visible cracks on the electrodes, and no obvious delamination between the electrode surface and copper foil was found by Gendensuren and Oh [66]. H-bond-based self-healing systems and their LIB applications have been summarized in Table 1.

Zhang et al. improved the self-healing ability of Si electrodes. Reconstruction of the crosslinked self-healable supramolecular polymer, which was facilely synthesized by copolymerization of tert-butyl acrylate and an ureido-pyrimidinone (PAA-UPy) monomer, followed by hydrolysis, is shown in Figure 4. A Si composite anode using a PAA-UPy binder gave an initial discharge capacity of 4194 mAh g^−1^ and a coulombic efficiency of 86.4% and maintained a high capacity of 2638 mAh g^−1^ after 110 cycles. The results reveal significant improvement of the electrochemical performance with PAA-UPy-based electrodes in comparison with that of Si anodes using conventional binders. It retained about 85% of the capacity in the long cycle. The self-healing PAA-UPy binder improved the electrochemical performance. They preferred using an ureidopyrimidinone-functionalized polyethylene glycol. This binder has important properties for resisting large volume expansion and healing cracks [40]. Zhang et al. reported a cyclic solid mesh binder for high-performance Si-based anodes. They took advantage of the interactions of cationic polyacrylamides (CPAM) and carboxymethyl cellulose (CMC). Electrodes prepared with this composite binder, as shown in Figure 4d,e, protected the electrochemical capacity of 1906.4 mAh g^−1^ after 100 cycles [67]. Hu et al. fabricated a self-healing gel by mixing PEDOT:PSS polymer and poly(vinyl alcohol). After modification with 4-carboxybenzaldehyde (CBA), hydrogen bonds were formed (Figure 4g). The half-cell of prepared electrodes with this binder showed a capacity of 1786 mAh g^−1^ at 500 mA g^−1^ after 200 cycles [35].

**Table 1:** Several self-healing materials for hydrogen bond interaction.

Self-Healing Material	Anode Active Materials	Electrolyte	Electrochemical Performance	Ref.
Na-alginate	Si-C composite anodes, 100 nm	1 M LiPF_6_ in EC/DEC/EMC	2850 mAh g^−1^ (first cycle capacity), 1250 mAh g^−1^ after 50 cycles at 0.1 A g^−1^	[60]
PAAH0.2Na0.8	Si/graphite < 100 nm	1 M LiPF_6_ in EC/DEC/EMC	1100 mAh g^−1^ after 30 cycles at 50 mA g^−1^,	[68]
Self-healing polymers (SHPs)	Silicon microparticle (SiMP)	1 M LiPF_6_ in EC/DEC/EMC	2617 mAh g^−1^ after 90 cycles at 0.4 A g^−1^	[62]
Carboxymethyl chitosan	Silicon, 100 nm	1 M LiPF_6_ in EC/DEC/EMC	950 mAh g^−1^ over 50 cycles at 500 mA g^−1^	[64]
Self-healing polymers (SHPs)	Silicon microparticle (SiMP), 1–3 µm	1 M LiPF_6_ in EC/DEC/VC/FEC	2736 mAh g^−1^ at C/20 after 500 cycles	[62]
Self-healing-type binder PAABS content binder 9 PAABS+6 CMC	Silicon 20–30 nm/graphite electrode	1 M LiPF_6_ in EC/DEC/EMC	1150 mAh g^−1^ (First cycle capacity), About 500 mAh g^−1^ after 50 cycles at 0.5 C	[69]
Native-XG, Na-CMC, alginate	Si/graphite, 100 nm Si	1 M LiPF_6_ in EC/DEC/EMC	14.2%, 22.8%, and 34.6% of capacities after 200 cycles at 1 C, respectively	[70]
Urea via hydrogen bonds	Silicon particles (~0.9 µm)	1 M LiPF6 in EC/DEC/VC/FEC	1700 mAh g^−1^; 80% retained after 175 cycles at C/20	[24]
Self-healing polymers (SHPs)	Silicon, 100 nm	1 M LiPF_6_ in EC/DEC	719 mAh g^−1^; 81% retained after 100 cycles at 1 C	[29]
Crosslinked chitosan (CS-GA)	Silicon, 100 nm	1 M LiPF_6_ in EC/DEC/EMC	1969 mAh g^−1^ after 100 cycles at 500 mA g^−1^	[71]
Pyrene–poly(acrylic acid)– polyrotaxane	Silicon	1 M LiPF_6_ in EC/DEC/EMC	82.5% retained after 150 cycles at 0.5 C	[40]
Dual-crosslinked network binder of alginate	Silicon/graphite anodes	1 M LiPF_6_ in EC/DEC/VC/FEC	1743 mAh g^−1^; 74% retained after 200 cycles at 2000 mA g^−1^	[66]
PAA-UPy	Silicon	1 M LiPF_6_ in EC/EMC/DMC	2000 mAh g^−1^; 74% retained after 110 cycles at 1 C	[40]
UPy-functionalized PEG	Silicon	1 M LiPF_6_ in EC/DMC	1847 mAh g^−1^; 81% ICE at 1 C	[72]
CMC-CPAM	Silicon-based	1 M LiPF_6_ in EC/DEC/EMC	2103 mAh g^−1^; 92% ICE at 2 C	[67]
PEDOT-PSS-PVA	Silicon	1 M LiPF_6_ in EC/DEC/EMC	1743 mAh g^−1^; 74% retained after 200 cycles at 2000 mA g^−1^	[35]
PAU-g-PEG,	Silicon	1 M LiPF_6_ in EC/DEC/EMC	2831 mAh g^−1^/3.2 mAh cm^−2^; 85% retained after 120 cycles at 0.1 C	[41]
Self-healing polymers (SHPs)	Silicon microparticle (SiMP)	1 M LiPF_6_ in EC/DEC/EMC	2100 mAh g^−1^; 91.8% capacity retention after 100 cycles at C/10	[63]

Table 1 provides comprehensive details on the self-healing materials with H-bonds used as electrodes, the cell system in which self-healing electrodes are used, and its electrochemical evaluation. According to Table 1, the Si@PAU-g-PEG electrode showed an outstanding electrochemical performance of over 2500 mAh g^−1^ over 100 cycles under a C ratio of 0.1C. A ureido-pyrimidinone (UPy)-functionalized poly(acrylic acid) grafted with poly(ethylene glycol)(PEG) showed electronic integrity at an electrode during repeated charge–discharge cycles in the self-healing mechanism-formed structure within molecules and via dynamic hydrogen bonds with silicon. It can be said that silicon effectively accommodated volume changes compared to other anodes in the table, which showed self-healing properties with hydrogen bonding. As seen in the table, while the electrochemical performance of carboxymethyl chitosan was close to 1000 mAh g^−1^, the Si anodes using the self-healing mechanism formed by carboxymethyl cellulose and the cationic polyacrylamides added polymer showed two times the electrochemical performance at 2C. To make a comparison between Si/C electrodes, the cell prepared from a self-healing structure with a dual-crosslinked network binder of alginate maintained excellent cyclability with a high capacity of approximately 1700 mAh g^−1^, even after one hundred cycles.

#### 2.1.2. Metal Interaction of Self-Healing Materials

**Figure 5:**
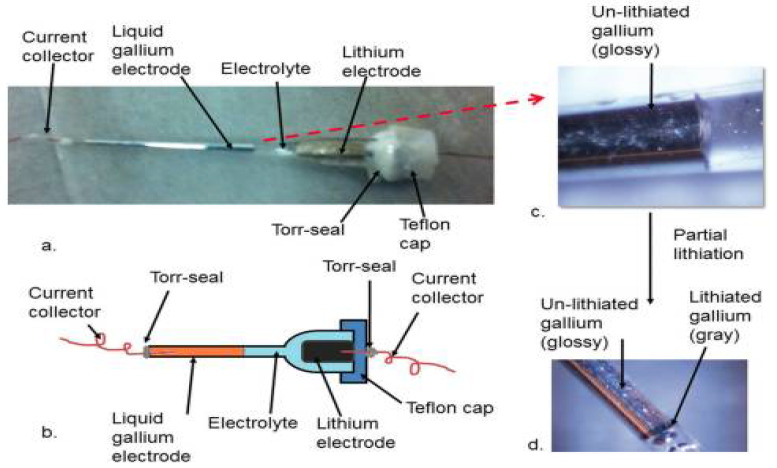
(**a**). A capillary cell with (**b**). its conceptual picture. (**c**). Magnified view of the surface near Ga electrode before lithiation and (**d**). after partial lithiation. Reprinted with permission from ref. [73], copyright 2011, Elsevier.

A self-healing mechanism occurs when a metal atom is attached to the side chains as part of the backbone or to the ends of the polymer chains or coordinated with covalently bonded ligands within the polymer backbone. Liquid metal gallium (Ga) was used for the first time by Deshpande et al. as a negative electrode with self-healing properties. It has been observed that low-melting-point liquid metals heal cracks during a reversible solid–liquid transition during charge–discharge [73]. Desphande et al. investigated the reversibility of lithiation of the LM pure Ga as a negative electrode for an LIB. Ga hosts two Li atoms per Ga atom upon full lithiation, delivers a theoretical gravimetric capacity of 769 mAh g^−1^ by forming Li_2_Ga alloy, and shows a discharge potential close to that of the Li/Li^+^ reaction. It has been shown that LiGa alloys, CuGa alloys, and Ga confined in a carbon matrix deliver capacities of about 200–400 mAh g^−1^ upon extended cycling. The capillary cell concept has been illustrated in Figure 5a–d. Glass capillary cells were prepared by assembling with liquid gallium as the working electrode and lithium metal as the counter electrode. An EC/DEC electrolyte was used. The capillary was filled with liquid Ga at one end, and solid lithium attached to a copper current collector was placed at the other end. The space between the electrodes was filled with the electrolyte. 

For the first time, Han et al. reported the production of a gallium–indium–tin (GaInSn) alloy Si composite anode was healed by covering the cracks after Ga particles damaged during cycling (Figure 5) [73].

Various and excellent studies in the literature have been summarized in Table 2. Anode active material from Ga to Si with different polymer binders showed very high capacities in a long cycle life.

**Figure 6:**
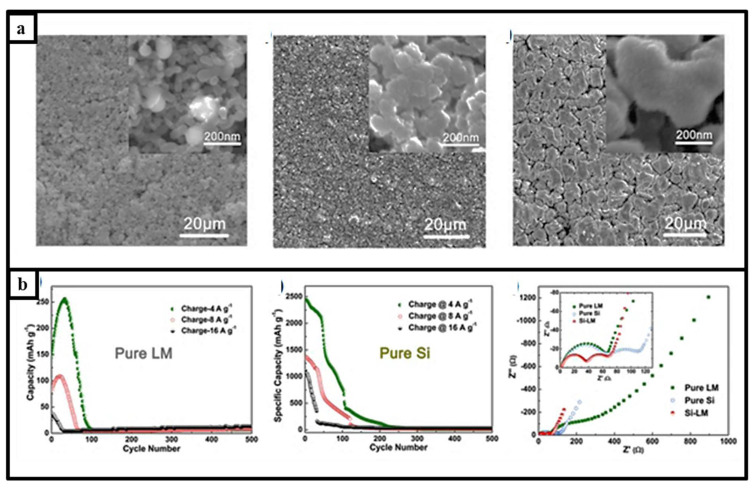
(**a**). Morphology of liquid metal–silicon electrode before the cycle, after the 200th cycle, and after the 1500th cycle. (**b**). Capacity–cycle graph of liquid metal anode and Si anode cycled using different current densities for 500 cycles; Nyquist graph of the liquid metal anode, liquid metal–silicon anode, and silicon anode. Reprinted with permission from ref. [48], copyright 2018, Elsevier.

As mentioned above, Bing et al. presented a novel approach involving a composite system to heal the damage of material which was prepared through liquid-metal (LM)-mediated spontaneous repairing of a conductive-additive-free Si anode for a Li-ion battery [48]. The as-prepared nanocomposite of LM/Si showed superior performances, as characterized by high capacity utilization (2300 mAh g^−1^ after 200 cycles, long-term stability (968 mAh g^−1^ after 1500 charge–discharge cycles) and high rate capability (360 mAh g^−1^ at 20 A g^−1^). The surface morphology of the electrode and a Nyquist graph of the liquid metal anode are given in Figure 6a and b, respectively. As shown in Figure 6b, LM and Si nanoparticles had good electrochemical performance together, and the overall cell impedance was lower in the Si-LM anode than in pure LM and pure Si [48].

**Figure 7:**
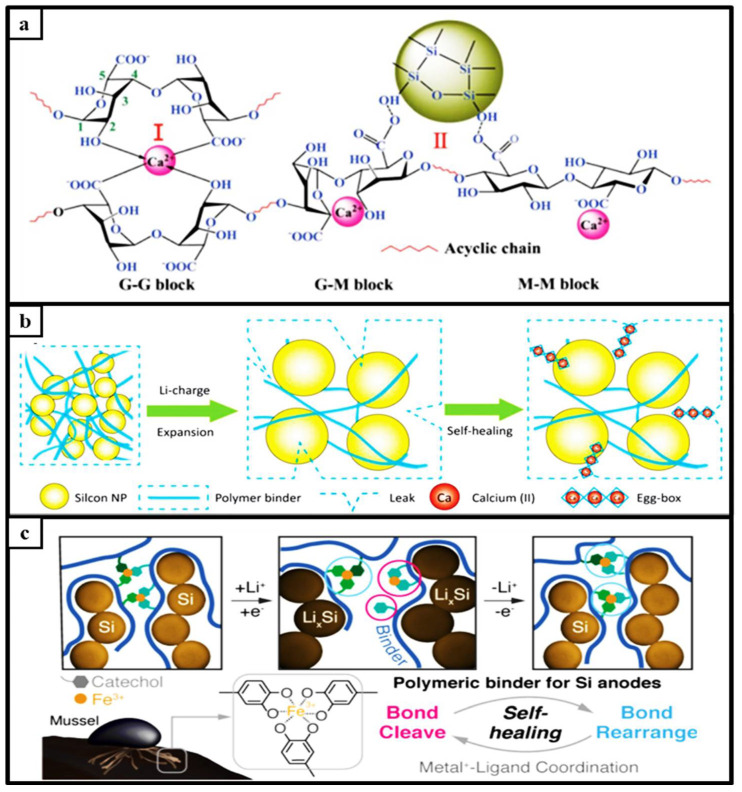
(**a**). Scheme of coordinate bonds between alginate chains and calcium cations, reprinted with permission from ref. [74], copyright 2014, Elsevier. (**b**). Self-healing illustration of Ca–alginate–silicon anode during cycling, reprinted with permission from ref. [75], copyright 2014, Elsevier. (**c**). Scheme of silicon–self-healing binder over Fe^+3^/cathecol–based bond cleave and rearrangement type (metal–ligand complex) healing system, reprinted with permission from ref. [76], copyright 2019, *Advanced Materials*.

Metals not only benefit from their low melting point temperature but also contribute to displaying reversible properties by coordinating with polymeric networks. It helps maintain electrical and mechanical integrity and significantly suppresses the volume expansion of the silicon anode by forming coordination bonds with the Ca^2+^ cations of the alginate chain self-healing mechanism with hydrogen bonds (Figure 7a). As a result, this structure showed a capacity of 2522 mAh g^−1^ with 76.5% capacity retention after 500 cycles [74]. Metals are used to coordinate more alginate chains with the alginate, which is expected to crosslink. The results show that Si anodes with Al-algae or Ba-algae binding are more robust and represent higher capacity retention with their reversible properties. Yoon et al. developed a simple Ca spray treatment method with a Ca^2+^-doped alginate Si anode composite. (Figure 7b). LIBs are anticipated to have a longer life and higher charge capacity than others. The Si-Ca electrodes have a 1711 mAh g^−1^ charge capacity at 0.2 A g^−1^, and even at a high current density of 2000 mA g^−1^ [75]. It has been shown that it gains self-healing properties with the coordination of Fe^3+^ (tris)catechol. An anode in this coordination structure exhibits 81.9% capacity retention after 350 cycles at 1C. Fe^3+^, and (tris)catechol is like a crosslinked polymeric network (Figure 7c) that gives it flexibility. A tridentate ligand structure, Fe^3+^ and (tris)catechol, gained flexibility [76].

Table 2 provides comprehensive details on the self-healing materials used as electrodes, the cell system in which self-healing electrodes are used, and its electrochemical evaluation. According to Table 2, the Si@ Alginate binder with Ca^2+^ ion electrodes showed an outstanding electrochemical performance of over 2500 mAh g^−1^ over 500 cycles under a C ratio of 20 C. The calcium-mediated electrostatic crosslinking of alginate improves the flexibility of the alginate binder and electrolyte desolvation. The improved mechanical properties of the calcium alginate binder compared to the sodium alginate binder overcome the barriers to volume expansion of silicon and increase the capacity of the Si anodes. For the discharge capacity, the Ga-In-Sn alloy electrode showed 2300 mAh g^−1^ at 0.25 C. The self-healing mechanism takes place at the low melting temperature of gallium. On the other hand, the electrochemical performance of liquid gallium, which has properties of self-healing with a solid–liquid transition, was observed to have a value close to the electrochemical performance of the gallium–silicon composite electrode.

#### 2.1.3. Host–Guest Interactions

**Figure 8:**
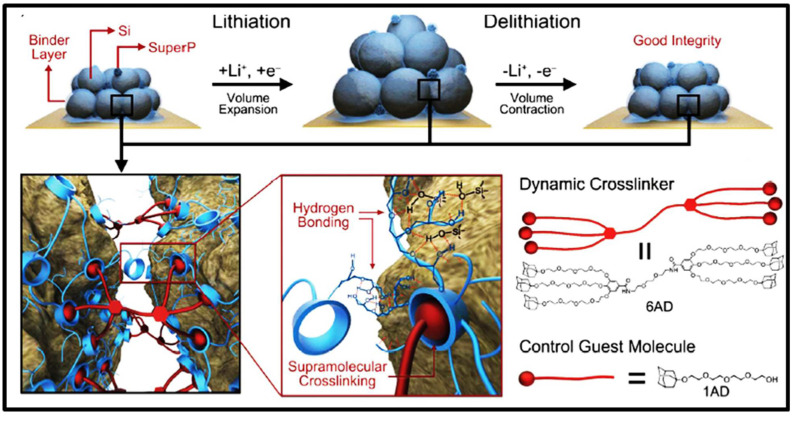
The mechanism of dynamic crosslinking for silicon nanoparticle anodes by engaging host–guest interactions between hyperbranched βcyclodextrin polymer and a dendritic-gallic-acid-derived supramolecular crosslinker incorporating six adamantane units. Reprinted with permission from ref. [78], copyright 2015, American Chemical Society.

Kwon et al. fabricated host–guest interactions with a hyperbranched β-cyclodextrin polymer, a hydrophobic guest, adamantine, a guest moiety with high affinity and selective binding to β-cyclodextrin, and a dendritic gallic acid crosslinker with six adamantine units as the host [78]. This system between the guest and host polymer chains provides crosslinking polymer binders for silicon anodes during volume variations, resulting in a 90% capacity retention after 150 cycles (Figure 8) [78]. Crosslinked hyperbranched β-cyclodextrin and a gallic-acid-based silicon anode with electrolytes of 1M lithium hexafluorophosphate solution in ethylene carbonate, diethyl carbonate, vinylene carbonate, and fluoroethylene carbonate (1M LiPF_6_ in EC/DEC/VC/FEC) achieved a 1500 mAh g^−1^ at 0.5 C cycling performance, and 90% was retained after 150 cycles.

#### 2.1.4. Ionically Bonded Interaction

**Figure 9:**
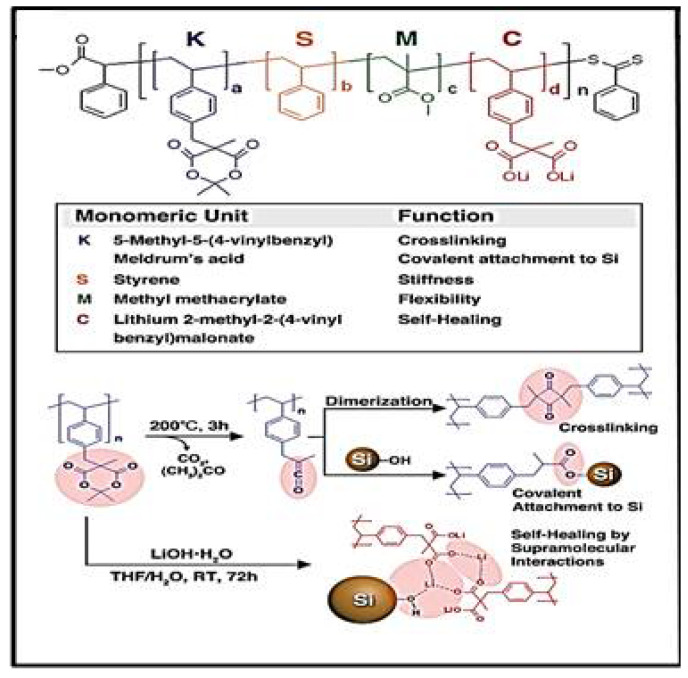
Chemical structures of polymers and synthesis of lithium 2-methyl-2-(4-vinylbenzyl) malonate from Meldrum’s acid, reprinted with permission from ref. [79], copyright 2014, *Advanced Materials*.

Polymeric materials with macromolecules consisting of ionic and/or ionizable groups can be developed that show the advantage of reversible physical crosslinks for self-healing functions, forming interactions not found in nonionic polymers. The ionic content can assist the diffusion/sealing process and stabilize the fracture resistance of the polymer material. Kwon et al. presented a material in which each monomeric group has a functionality in the main chain, ranging from stiffness, cross-linking, and flexibility to self-healing. Lithium 2-methyl-2-(4-vinylbenzyl)malonate can be prepared in one step as seen in Figure 9 upon hydrolysis of Meldrum’s acid for a self-healing effect via ion–dipole interactions between polymers and also with the native silanol groups on the Si surface [79].

The cycling performance of polymer composites and silicon achieved 51% capacity retention after 500 cycles [79]. Wu et al. created a self-healing porous scaffold structure by exploiting the electrostatic interaction between the carboxylate (–COO^−^) of Alg and the protonated amines (–NH^3+^) of C-chitosan in the alginate–carboxymethyl–chitosan (Alg-C-chitosan) composite polymer. A Si-based anode using an Alg-C-chitosan composite binder exhibited excellent cycle stability with a residual capacity of 750 mAh g^−1^ after the 100th cycle [20].

The polymer binder found by Zeng et al. shows 14- and 90-times higher lithium-ion dispersion and electron conductivity, respectively, than the commonly used carboxymethyl cellulose and acetylene black. When prepared from this ionic (polyethylene oxide and polyethylenimine) polymer binder, the silicon anode had a high capacity of approximately 2000 mAh g^−1^ at 500 cycles at 1C [67]. Huang et al. developed a self-healing ionomer-electrode that presented a better initial discharge areal capacity of 2.9964 mAh cm^−2^ compared with 2.9748 mAh cm^−2^, 2.895 mAh cm^−2^, 2.991 mAh cm^−2^ at a rate of 0.1 C for electrodes with PVDF, SBR, and uncrosslinked ionomer binders, respectively [11]. Several self-healing materials for ionically bonded interactions have been summarized in Table 3.

Table 3 provides comprehensive details on the self-healing materials used as electrodes, the cell system in which self-healing electrodes are used, and its electrochemical evaluation. The silicon anode with the polymer binder had a high reversible capacity of over 2000 mA h g^−1^ after 500 cycles at a current density of 1.0 A g^−1^, while, as seen in Table 1, the electrochemical performance of carboxymethyl chitosan was close to 1000 mAh g^−1^. With the addition of alginate to CMC, the self-healing properties of the structure may be reduced due to hydrogen bonds with an ionic effect.

#### 2.1.5. Multiple Functional Interactions of Self-healing Mechanism

**Figure 10:**
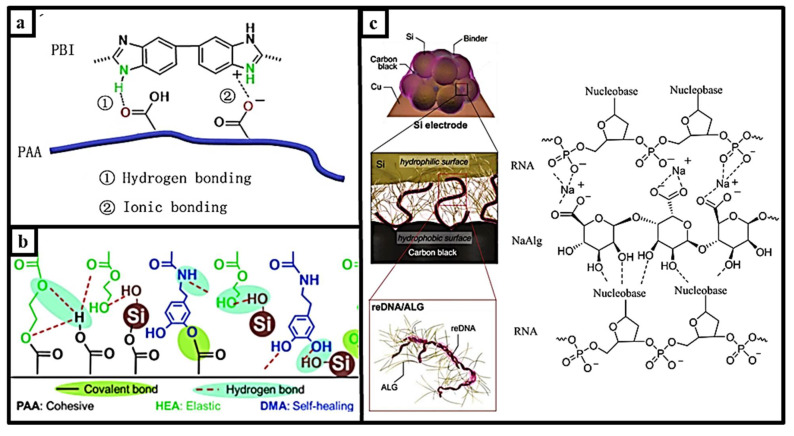
(**a**). The chemical structure of PAA-PBI, reprinted with permission from ref. [81], copyright 2015, American Chemical Society. (**b**). Self-healing interaction with silicon and binder, reprinted with permission from ref. [15], copyright 2018, Elsevier. (**c**). The interactions between binder, silicon particles, and CB and the structure of reDNA/alginate, reprinted with permission from ref. [82], copyright 2018, *Advanced Materials*.

Self-healing properties can be intramolecular and intermolecular, as well as a self-healing material with physical interaction combined with chain movements and multilevel chemical interactions obtained by repairing more than one type of chemical entity in a single material. Lim et al. reported poly(acrylic acid) (PAA)—poly(benzimidazole) (PBI) binding using a supramolecular interaction with an ionic bond and a hydrogen bond. This highlights that the structure using only the PAA binder with 0.45 peeling and the structure using 2% by weight PBI relative to PAA show close mechanical properties. Thus, it shows that a tight conducting network (Figure 10a) was obtained using PAA-PBI-2. This mechanical property is related to the proportional reversibility of hydrogen bonding and ionic interactions. The electrode with the PAA-PBI-2 connector showed a high initial capacity of 1376.7 mAh g^−1^ and improved capacity retention of 54.6% after 100 cycles, which was much better than the other two connectors. The bond strength of the bonding network with Si will decrease with an increasing PBI ratio because the ionic interaction between PBI and PAA provides a lower amount of carboxylic acid to adhere to the Si surface [81]. The cycle performance of the electrodes can be improved with two or more different types of dynamic bonds in a well-designed binder. Xu et al. showed that a polymer that has multiple interactions of poly(acrylic acid)-poly(2-hydroxyethyl acrylate-co-dopamine methacrylate) (Figure 10b) was prepared by mixing PAA with P(HEAco-DMA). Kim et al. reported a polymer linker composed of DNA (reDNA) and NaAlg using two supramolecular interactions. This supramolecular interaction suppresses the volume change in the Si electrode by physically crosslinking the hydrogen bond and the ionic bond, which has been illustrated in Figure 10c. The Si/reDNA/NaAlg electrode exhibited a capacitance efficiency of 80.1% after 160 cycles at a current density of 1.75 Ag1, while the blank trials Si/reDNA, Si/DNA, and Si/NaAlg electrodes achieved efficiencies of 67.8%, 61.3%, and 48.6%, respectively [82]. An extremely stable cycle life was demonstrated with the PAA-connected electrode alone, which showed a much faster capacitance drop over 100 cycles than this combined polymer [15]. Several self-healing materials for multiple functional interactions have been summarized in Table 4.

Table 4 provides comprehensive details on the self-healing materials used as electrodes, the cell system in which self-healing electrodes are used, and its electrochemical evaluation. According to Table 4, the Si@ PAA-P(HEA-*co*-DMA) electrode showed an outstanding electrochemical performance of over 1800 mAh g^−1^ over 160 cycles at 1.75 A/g^−1^. The high content of hydrogen bonding sites and the covalent structure with catechol groups provided some self-healing capability to the flexible poly(acrylic acid)-poly(2-hydroxyethyl acrylate-*co*-dopamine methacrylate) polymer. Thus, the cycle stability and speed performance of the Si anode was significantly improved.

### 2.2. Chemical-Interaction-Based Self-Healing Materials

The reversibility of covalent bonds can use condensation, exchange, and addition reactions. For the first time, Kim et al. developed self-healing processed by a thermoplastic polyurethane (TPU) designed by easy-to-process aromatic disulfides that can properly self-heal within 2 h through aromatic disulfide metathesis [82]. Xu et al. was inspired by nature to prepare a new poly(urea-urethane)–graphite carbon nitride nanolayer composite material in which multiple hydrogen bonds in the PUU matrix impart graphitic carbon nitride with self-healing ability at room temperature to the composite material. Improved mechanical properties of the composite material are provided by nanolayers that serve as both chemical and physical crosslinkers [83]. Li et al. reported a supramolecular polymer type using a host–guest complex of visible-light labile picolinium β-cyclodextrin nanogels (β-CD) with ultrastability against electrolytes and photodegradation properties [27]. Most self-healing artificial materials are polymer-based [17]. Self-healing mechanisms can be classified in various ways according to the way in which bonds are broken and joined and the nature of intramolecular and intermolecular interactions, external excitations, and polymer network structures. In its simplest form, it has two main types: covalent and noncovalent. Self-healing mechanisms with dynamic noncovalent bonds are hydrogen bonding, ionic interactions, metal coordination, π-π stacking, and hydrophobic interactions, while self-healing mechanisms with dynamic covalent bonds include the Diels–Alder reaction, disulfide, acylhydrazone, ester, and imine. The Diels–Alder (DA) reaction for crosslinking linear polymers has been pioneered by Kennedy and Wagener over the last four decades [84,85]. The reversible groups of the thermo-reversible polymers were attached to linear polymer backbones, but the links of crosslinkers to polymer backbones were not reversible, using a completely reversible covalently formed macromolecular network, as reported by Chen et al. [86]. A mechanically self-healing electrode was successfully developed by Lee et al. with combining Ag nanowires and polydimethylsiloxane-based polyurethane (PDMS-CPU) crosslinked with Diels–Alder (DA) adducts. A combination of the DA reaction with coated AgNWs on the surface of the polymer smoothed the polymer surface, greatly improving the mechanical sustainability of the electrode’s surface [87]. Similarly to this work, a transparent electrode, a thermally replaceable electrode, was developed by Pyo et al., again using polyurethane Ag nanowires as crosslinkers [88].

As an alternative to the self-healing chemistry of covalently bonded rubber materials, the disulfide mechanism has been used [89]. Disulfide groups can be cleaved by a reduction reaction to form two thiol groups. Then, it can be regenerated by oxidation. Tesoro et al. reformed epoxy resins with a disülfide interaction [90,91]. Tobolsky et al. showed the change in sulfur–sulfur bonds in poly(ethylene disulfide) and poly(ethylene tetrasulfide), as well as polyurethanes [92,93]. Finally, thiol-terminated poly(styrene) synthesized via a disulfide interaction during reduction–oxidation was reported by Tsarevsky et al. [92]. Kuhl et al. studied self-healing polymer networks obtained by polymerization of an acylhydrazone crosslinker and methacrylates to improve the mechanical properties of the polymer by adjusting the Tg [93]. Uncrosslinked polymers and those with reversible crosslinks can be processed but are soluble. Leibler and colleagues demonstrated the reprocessability of epoxy acid network polymers at high temperatures with ester bonds in a covalent network. [94]. Yu et al. showed, with thermally malleable polymers that undergo covalent bond esterification exchange reactions, that the glass transition temperature of the material increases as the proportion of hard segments in the epoxy increases [95]. Imine chemistry, also known as Schiff base chemistry, involves reversible covalent interaction [96]. Chao et al. showed that the imine bond exchange is induced by residual primary amino functionalities in the polymeric network [97].

#### 2.2.1. Imine-Bond-Based Self-Healing Systems

**Figure 11:**
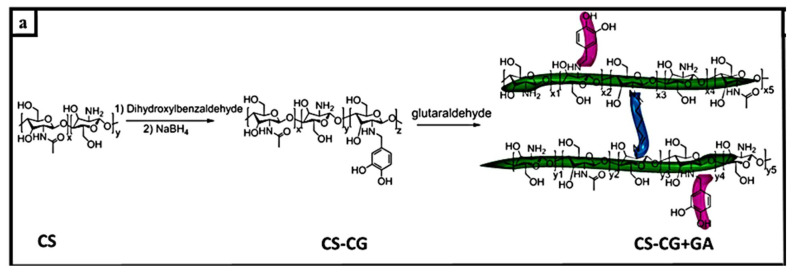

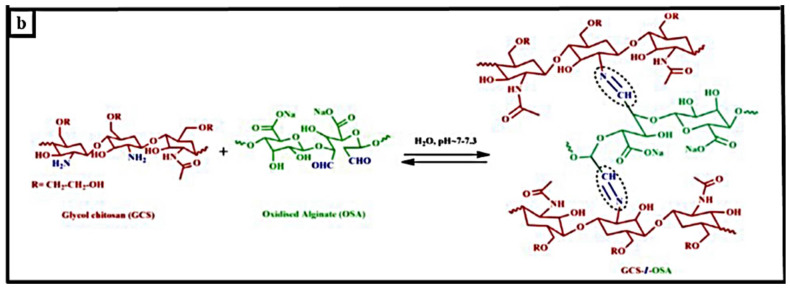
(**a**). Synthesis of a crosslinking catechol-rich network, reprinted with permission from ref. [98], copyright 2019, American Chemical Society. (**b**). The self-healing process between glycol chitosan and oxidized alginate (OSA) and schematic comparison of GCS-I-OSA binder and traditional binder, reprinted with permission from ref. [34], copyright 2020, Elsevier.

According to Cao et al., catechol-functionalized chitosan crosslinked with glutaraldehyde (CS – CG + GA) (Figure 11a), a SiNP-based anode with polymer mesh (CS-CG 10% + 6%GA) via catechol grafting, showed a capacity retention of 91.5% after 100 cycles at 2144 ± 14 mAh g^−1^ [98]. Rajevv et al. showed a self-healing network formed between amino groups in glycol chitosan and aldehyde groups (Figure 11b). These components include a dynamic Schiff base reaction. Si electrodes (GCS-I-OSA) exhibited a high degree of reversibility of 2316 mAh g^−1^ at 0.2 C after 100 cycles, while Si/graphite composite anodes showed a current density of 0.2 C after 100 cycles. It exhibited a specific capacity of 1364 mAh g^−1^ [34]. Several self-healing materials for imine-bonded interactions have been summarized in Table 5.

Table 5 provides comprehensive details on the self-healing materials used as electrodes, the cell system in which self-healing electrodes are used, and its electrochemical evaluation. According to Table 5, the Si electrode with catechol-functionalized chitosan crosslinked by glutaraldehyde showed an outstanding electrochemical performance of over 2100 mAh g^−1^ over 100 cycles at 1 C. Gutaraldehyde (CS – CG + GA), serving dual functions, was crosslinked with the polymer binder, i.e., chitosan with a catechol function. It is advantageous with its wet-resistant adhesion through catechol grafting and mechanical strength through the in situ formation of a three-dimensional structure and offers a high capacity by preventing volume expansion of silicon.

#### 2.2.2. Ester-Bond-Based Self-Healing Systems

Ryu et al. investigated the natural guar gum component BC g (boronic crosslinked guar) on Si anode. This binder, which will maintain the electrode integrity over long cycles, adheres strongly to the surface of the Si particles with its hydroxyl content. In the polymer, the bonding between the boronic acid side groups on the polystyrene backbone and the hydroxyl groups on the guar gum increases the mechanical strength. Hydroxy H-bonds and borate ester bonds form the self-healing mechanism. By putting a drop of electrolyte solvent on the broken surfaces, the polymer was able to reconnect the new surfaces. The prepared Si electrode containing the developed polymer binders retained 70% capacity after 300 cycles at 1C [34].

**Figure 12:**
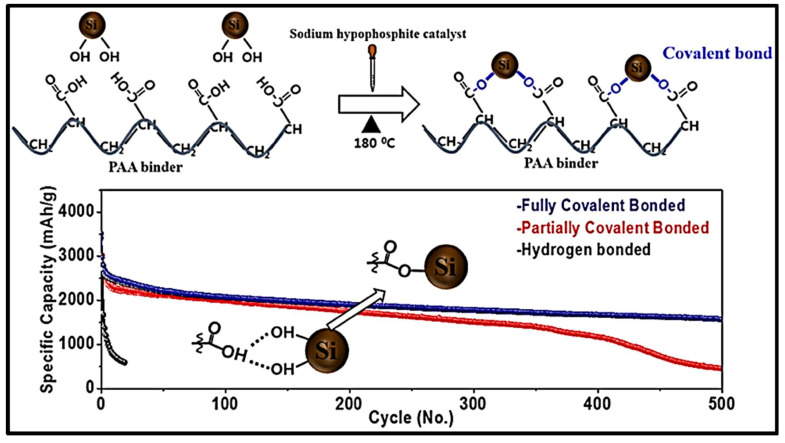
Cycle–capacity of the different types silicon electrodes and the interactions between Si nanoparticles and PAA binder, reprinted with permission from ref. [99], copyright 2019, American Chemical Society.

Jung et al. improved a Si anode which has properties stabilizing the SEI layer and preventing the volumetric expansion of Si aggregation (Figure 12). Jung et al. designed a binding approach that enables covalent bond formation between −OH groups of the polyacrylic acid with Si’s surface. This combination exhibited a capacity of 1500 mAh g^−1^ after 500 cycles at 1000 mA g^−1^ [99]. Several self-healing materials for boronate ester bonds have been summarized in Table 6.

Table 6 provides comprehensive details on the self-healing materials used as electrodes, the cell system in which self-healing electrodes are used, and its electrochemical evaluation. According to Table 6, the Si electrode with esterificated PAA showed an outstanding electrochemical performance of over 1500 mAh g^−1^ over 500 cycles at 1 A g^−1^. Silicon anode prepared with polyacrylic acid binder consisting of silicon and −COOH groups treated with piranha solution to produce −OH reached a self-healing structure with the effect of an effective ester bond and offered a high capacity in long-cycle stability.

#### 2.2.3. Disulfide-Bond-Based Self-Healing Systems

It affects the molecular behavior of the types and steric hindrance of self-healing fragments between different polymer chains and can self-heal and has mechanical properties. The urea groups are self-healing due to differences between the thiourea and urea hydrogen bond moieties.

**Figure 13:**
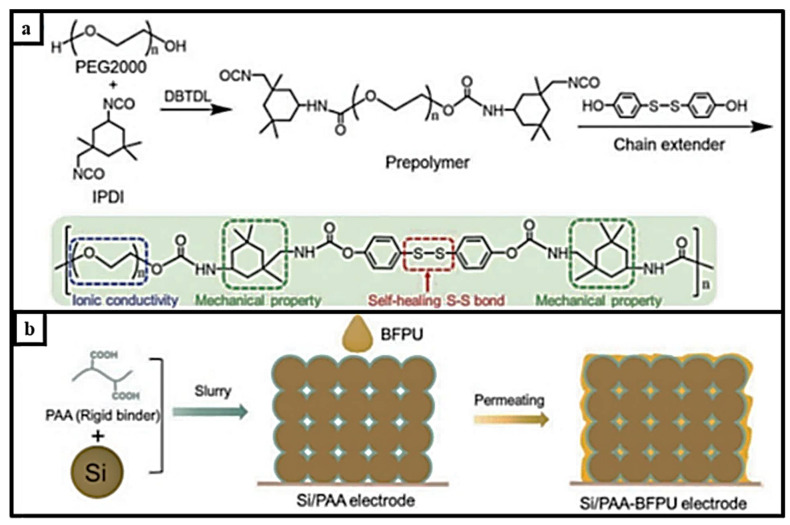
(**a**). The BFPU polymer that has different functionalities, from ionic conductivity and mechanical durability to self-healable S-S bonds (**b**). Silicon electrode with PAA–BFPU binder, reprinted with permission from ref. [101], copyright 2021, *Advanced Materials*.

A double-wrapped binder polyacrylic acid (PAA) and binder using outer polyurethane (BFPU) polymers (Figure 13a) to address the large internal stress silicone were developed by Jiao. BFPU acts as a buffer layer to disperse the internal tension and stress during lithiation. This prevents structural damage to the hard PAA. Thus, large volume changes are prevented during the charge–discharge process. Si anodes developed with a PAA–BFPU binder, as can be seen in the illustration in Figure 13b, had a capacity of 3.5 mAh cm^−1^ and over 88% capacity retention for 200 cycles [100]. Several self-healing materials for disulfide-bonded interactions have been summarized in Table 7.

Table 7 provides comprehensive details on the self-healing materials used as electrodes, the cell system in which self-healing electrodes are used, and its electrochemical evaluation. According to Table 7, the Si electrode 1,6-bismaleimide-(BMI) functionalized poly(acrylic acid) (FPAA) DA-PAA showed an outstanding electrochemical performance of over 1000 mAh g^−1^ over 200 cycles at 1 C. The PAA binders, which have self-healing properties with a disulfide bond, as shown in Table 7, did not yield a better result than silicone anode prepared with a polyacrylic acid binder.

#### 2.2.4. Diels–Alder-Reaction-Based Self-Healing Systems

**Figure 14:**
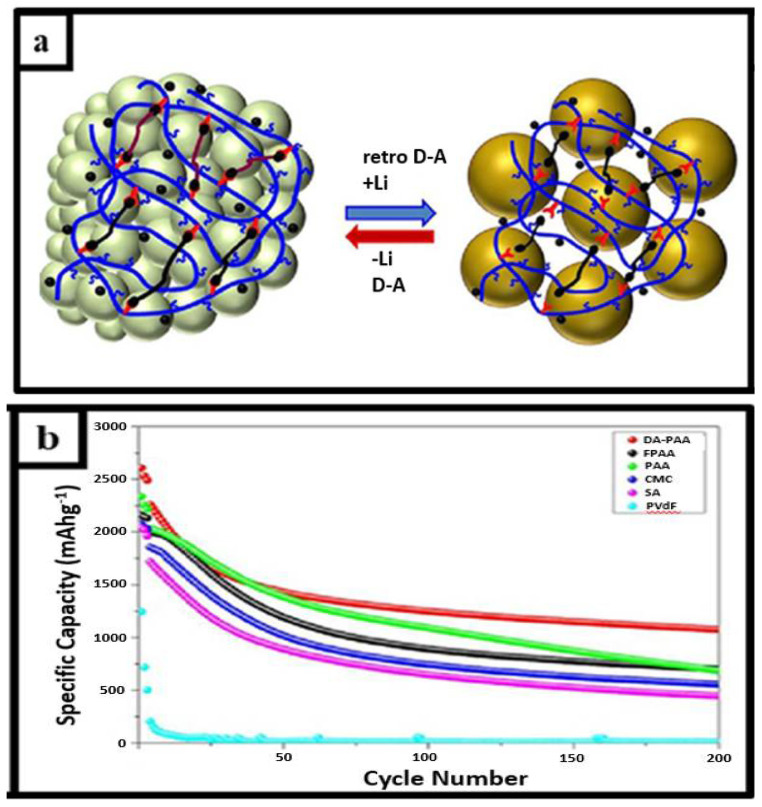
(**a**). Diels-Alder chemistry in silicon anode with self-healing binder (**b**). The comparison of cycle-capacity among binders, reprinted with permission from ref. [22], copyright 2021, *Advanced Materials*.

Rajeev et al. reported creating a crosslinked polymer network based on 1,6-bismaleimide (BMI) as a crosslinker for furfurylamine-functional poly(acrylic acid) (FPAA) and then thermal Diels–Alder (DA) click chemistry, which was used as a new polymer for silicon binding. The crosslinked network of Diels–Alder chemistry exhibited self-healing with Diels–Alder chemistry. The Si electrode with the Diels–Alder binder recorded a high Coulombic efficiency of 99.7% after 200 cycles. The Diels–Alder-PAA binder shown in Figure 14a outperformed commercially available silicone binders, such as PAA, CMC, SA, and PVdF, which were compared in terms of cyclic performances, the results of which can be seen in Figure 14b [22].

## 3. Conclusions

Self-healing materials have been extensively researched, from electronics to the building industry and the biomedical fields. In addition to these areas, the application of self-healing materials to electrochemical-based devices, especially lithium-ion batteries or supercapacitors, is rapidly increasing. Many studies have been conducted on the manufacture of other electronic and electrochemical devices, such as dielectric actuators and electrochemical sensors concerning self-healing systems. Conductive polymers are very important for solar cells, actuators, sensors, batteries, and other energy storage devices. However, damage to these materials causes serious problems in device performance. The main requirement for the development of self-healing and conductive materials is to maintain a high conductivity level after damage. One of the most basic strategies for preparing self-healing conductive materials is to add dynamic reversible bonds to the structure.

The “self-healing with smart modifications” approach, which has attracted increasing attention from the scientific community around the world, gives priority to systems that allow the use of new-generation polymeric binders in silicon anodes. Therefore, the self-healing systems in silicon anodes for LIBs have increased in the past few years. A comprehensive review of self-healing systems in silicon anodes for LIBs has been prepared with this approach. In this regard, novel researches about self-healing systems in silicon anodes for LIBs have been gathered to present the most recent advances to battery society. We hope that the self-healing systems in silicon anodes for LIBs will be applied for novel scientific approaches in near future.

In conclusion, although promising developments have been achieved so far, innovative materials strategies are still needed at the application level of self-healing materials and tools for practical use and eventual commercialization.

## Figures and Tables

**Table 2:** Several self-healing electrode examples over metal interaction.

Self-Healing Properties	Anode Active Material	Electrolyte	Electrochemical Performance	Ref.
Solid–liquid transition of gallium	Liquid gallium	1M LiPF_6_ EC/DEC/DMC	626 mAh g^−1^ at C/5	[73]
Alginate binder with Ca^2+^ ions	Silicon sub-microparticule 200 nm	1M LiPF_6_ EC/DEC/DMC (1:1:1 volume ratio)	2522 mAh g^−1^ after 500 cycles at 20 C	[74]
Ca-alginate binder	Silicon, 100 nm	1.3 M LiPF_6_ EC/EMC, 3:7 10wt% FEC	1711 mAh g^−1^ at 0.2 A g^−1 1^ after 300 cycles	[75]
Low melting temperature	Ga-In-Sn alloy	1M LiPF_6_ in EC/EMC/DMC	2300 mAh g^−1^ at 0.25 C	[48]
Fe-ß-catechol coordination bonds	Silicon microparticule. 300 nm	1M LiPF_6_ in DMC/EC	81.9% capacity retention after 350 cycles at 1 C	[76]
The lithiation–delithiation mechanism	Liquid CuGa_2_/Si nanocomposite	1M LiPF_6_ in EC/DEC	630 mAh g^−1^ at 200 mA g^−1^	[77]

**Table 3:** Self-healing materials for ionically bonded healing mechanism.

Self-Healing Material	Anode Active Materials	Electrolyte	Electrochemical Performance	Ref.
Meldrum’s acid	Silicon	1M LiPF_6_ in EC/DEC	1743 mAh g^−1^; 74% retained after 200 cycles at 2000 mA g^−1^	[79]
Alg–C-chitosan	Silicon	1M LiPF_6_ in EC/DEC	750 mAh g^−1^ after the 100th cycle	[20]
Ionic polymer with PEDOT:PSS, PEO, and PEI	Silicon	1M LiPF_6_ EC/DEC (1:1 volume ratio with 5% FEC	over 2000 mAh g^−1^ after 500 cycles at 1.0 A g^−1^	[80]
Content of binder/ conductive additive (%) 1.8/8	Si/graphite	1M LiPF_6_ EC/DEC/EMC (2:3:1 volume ratio)	71.7% capacity retention after 100 cycles at 0.5 C	[11]

**Table 4:** Several self-healing materials for multiple functional interactions mechanisms.

Self-Healing Material	Anode Active Materials	Electrolyte	Electrochemical Performance	Ref.
PAA-PBI with H-bond–ionic bond	Silicon, 100 nm	1M LiPF_6_ in EC/DEC	1376.7 mAh g^−1^ and improved capacity retention of 54.6% after 100 cycles at 1 C	[81]
reDNA/NaAlg Hydrophobic interaction	Silicon, 50 nm	1M LiPF_6_ in EC/DEC	80.1% capacity retention after 160 cycles at 1.75 A g^−1^	[82]
PAA-P (HEAco-DMA) H bond–covalent bond	Silicon, 50–100 nm	1M LiPF_6_ EC/DEC	1855 mAh g^−1^ under Si loading of 1 mg cm^−2^ at 5 A g^−1^	[15]

**Table 5:** Several self-healing materials for imine-bonded interactions of the healing mechanism.

Self-Healing Material	Anode Active Materials	Electrolyte	Electrochemical Performance	Ref.
Catechol-functionalized chitosan crosslinked by glutaraldehyde	Silicon nanoparticle, 100 nm	1M LiPF_6_ in EC/EMC	2144 ± 14 mAh g^−1^; 91.5% capacity retention after 100 cyclesat 1 C	[98]
GCS-I-OSA	Silicon powder 50 nm	1M LiPF_6_ in EC/DEC	1364 mAh g^−1^ after 100 cycles at 0.2 C	[34]

**Table 6:** Several self-healing materials for boronate ester of healing mechanism.

Self-Healing Material	Anode Active Materials	Electrolyte	Electrochemical Performance	Ref.
BC-g	Silicon powder 50 nm	1.3 M LiPF_6_ in DEC/EC	2750 mAh g^−1^; 87.3% capacity retention after 100 cycles at 0.2 C	[34]
Esterificated PAA	Silicon powder 50 nm	1M LiPF_6_ in DEC/EC/DMC 1:1:1 10% FEC	1500 mAh g^−1^ after 500 cycles at 1000 mA g^−1^	[99]

**Table 7:** Some self-healing materials for disulfide-bonded interactions of healing mechanism.

Self-Healing Material	Anode Active Materials	Electrolyte	Electrochemical Performance	Ref.
1,6-bismaleimide (BMI) functionalized poly(acrylic acid) (FPAA) DA-PAA	Silicon powder 50 nm	1 M LiPF_6_ in EC/EMC 1:2 (*v*/*v*) with 10% FEC	1076 mAh g^−1^; 99.7% capacity retention after 200 cycles at 1 C	[22]
Poly(ether-thioureas)	Silicon powder, 100 nm	1 M LiPF_6_ in DEC/EC/DMC	1325 mAh g^−1^ at 1 C	[100]
PAA–BFPU binder	Silicon powder, 100 nm	1 M LiPF_6_ in DEC/EC/DMC	over 88% capacity retention after 200 cyclesat 3.5 mAh cm^−2^	[101]

## Abbreviations

AMPS	2-Acrylamido-2-methylpropanesulfonic acid
AN	acrylonitrile
BA	acrylic acid n-butyl ester
CBA	4-carboxybenzaldehyde
CE	Coulombic efficiency
CMC	carboxymethyl cellulose
CPAM	cationic polyacrylamides
DC	discharge capacity
DEC	diethyl carbonate
DMC	dimethyl carbonate
EC	ethylene carbonate
EMC	ethyl methyl carbonate
FEC	fluoroethylene carbonate
Ga-In-Sn	gallium–indium–tin alloy
IC	ionic conductivity
ICE	initial coulombic efficiency
LiPF_6_	lithium hexafluorophosphate
LM	liquid metal
PEDOT	poly (3, 4-ethylene- dioxythiophene)
PSS	poly (styrenesulfonate)
PEGMA	poly (ethylene glycol) methyl ether methacrylate
PAA	poly(acrylic acid)
PVA	polyvinyl alcohol
PEG	polyethylene glycol
PEGA	poly (ethylene glycol) methyl ether acrylate
RT	room temperature
UPy	ureido-pyrimidinone
UPyMA	(2-(3-(6-methyl-4-oxo-1,4-dihydropyrimidin-2-yl)ureido)ethyl methacrylate)
VC	vinyl carbonate
